# Triple Reassortant H3N2 Influenza A Viruses, Canada, 2005

**DOI:** 10.3201/eid1207.060268

**Published:** 2006-07

**Authors:** Christopher W. Olsen, Alexander I. Karasin, Suzanne Carman, Yan Li, Nathalie Bastien, Davor Ojkic, David Alves, George Charbonneau, Beth M. Henning, Donald E. Low, Laura Burton, George Broukhanski

**Affiliations:** * University of Wisconsin-Madison, Madison, Wisconsin, USA;; † Animal Health Laboratory, Guelph, Ontario, Canada;; ‡ Canadian Science Centre for Human and Animal Health, Winnipeg, Manitoba, Canada;; § Ontario Ministry of Agriculture, Food, and Rural Affairs, Guelph, Ontario, Canada;; ¶ Swine Services Group, Stratford, Ontario, Canada;; # Ontario Ministry of Health and Long Term Care, Clinton, Ontario, Canada;; ** Ontario Ministry of Health and Long Term Care, Toronto, Ontario, Canada

**Keywords:** influenza A virus, porcine, swine, turkey, human, zoonoses, phylogeny, dispatch

## Abstract

Since January 2005, H3N2 influenza viruses have been isolated from pigs and turkeys throughout Canada and from a swine farmer and pigs on the same farm in Ontario. These are human/classical swine/avian reassortants similar to viruses that emerged in US pigs in 1998 but with a distinct human-lineage neuraminidase gene.

Influenza viruses of the classical H1N1 lineage were the dominant cause of influenza among North American pigs for >60 years (*1*). However, in 1998, H3N2 viruses emerged and rapidly spread throughout the US swine population (*2**–**4*). These were unique triple reassortant genotype viruses, with hemagglutinin (HA), neuraminidase (NA), and RNA polymerase (PB1) genes of human influenza virus lineage; nucleoprotein (NP), matrix (M), and nonstructural (NS) genes of classical swine virus lineage; and RNA polymerase (PA and PB2) genes of North American avian virus lineage. Further reassortment between these viruses and classical H1N1 swine viruses led to the emergence of reassortant H1N2 and H1N1 viruses among pigs in the United States (*1*). The reassortant H3N2 and H1N2 viruses have also been isolated from turkeys and ducks in the United States (*5**–**8*). Despite geographic proximity and cross-boundary trade in pigs and turkeys between the United States and Canada (9, D. Harvey, pers. comm.), these reassortant viruses did not initially infect animals in Canada. However, beginning in approximately January 2005, H3N2 influenza viruses swept rapidly across Canada. We describe the genetic characterization of reassortant H3N2 viruses from pigs, turkeys, and a swine farm worker in contact with sick pigs during this outbreak.

## The Study

Influenza viruses were isolated in Madin-Darby canine kidney cells from lung tissue or nasal swab samples from pigs of various ages (young growers to adults) manifesting influenzalike illness (ILI) in Manitoba in January (A/Swine/Manitoba/12707/05), Alberta in February (A/Swine/Alberta/14722/05), British Columbia in May (A/Swine/British Columbia/28103/05), and Ontario in July (A/Swine/Ontario/33853/05). No clear epidemiologic links existed between these farms. A/Ontario/RV1273/05 was isolated in primary rhesus monkey kidney cells from a nasal swab specimen collected as part of a diagnostic workup from an otherwise healthy farm worker in Ontario in whom ILI developed 2–3 days after onset of ILI among pigs on his premises. Fourteen-day courses of oseltamivir therapy were prescribed for this patient beginning the day he saw his physician and for 11 other potentially exposed farm workers beginning 2 days later. The patient recovered uneventfully; no other respiratory viruses were identified from his samples. A/Turkey/Ontario/31232/05 was isolated in embryonated hen's eggs from a cloacal swab sample from turkeys showing a severe drop in egg production. The turkey farm was located across the road from a swine farm at which pigs concurrently exhibited ILI, although virus isolates were not available from those pigs.

Nucleotide sequences of the full-length coding regions of all 8 RNA segments from each virus were determined by direct cycle sequencing with previously described techniques and primers (*3**,**10**,**11*). Related reference viruses were identified by BLAST (basic local alignment search tool) analyses, sequence comparisons were conducted by using DNASTAR software, version 6.3 (DNASTAR Inc., Madison, WI, USA), and phylogenetic relationships were estimated from the nucleotide sequences by the method of maximum parsimony (fast heuristic search algorithm, PAUP software, version 4.0b10 [Sinauer Associates, Inc., Sunderland, MA, USA]) with a bootstrap resampling method (200 replications).

**Figure 1:**
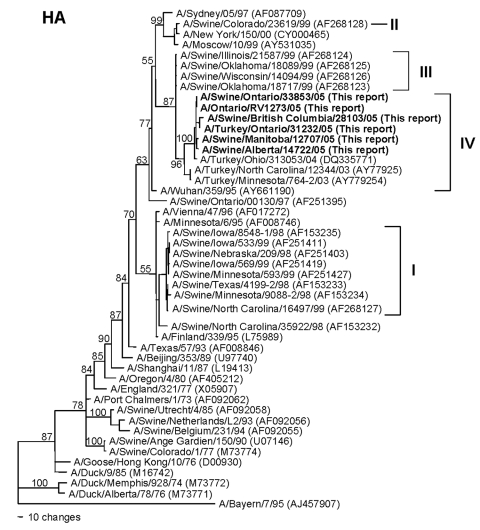
Nucleotide phylogram for the hemagglutinin (HA) genes of A/Swine/Manitoba/12707/05, A/Swine/Alberta/14722/05, A/Swine/British Columbia/28103/05, A/Swine/Ontario/33853/05, A/Turkey/Ontario/31232/05, A/Ontario/RV1273/05, and related reference viruses. The evolutionary relationships among these viruses were estimated by the method of maximum parsimony (PAUP software, version 4.0b10) with a bootstrap resampling method (200 replications) and a fast heuristic search algorithm. Numbers at the nodes of the phylograms indicate the bootstrap confidence levels. Horizontal-line distances are proportional to the minimum numbers of nucleotide changes needed to join nodes and gene sequences. Vertical lines are present to space the branches and labels. The designations I, II, and III identify clusters of viruses previously defined among triple reassortant swine viruses in the United States (*4*). The viruses described in this report and related viruses are proposed to represent a new cluster IV group of viruses. GenBank accession numbers for the sequences of all reference viruses are provided in parentheses after the virus names.

**Figure 2:**
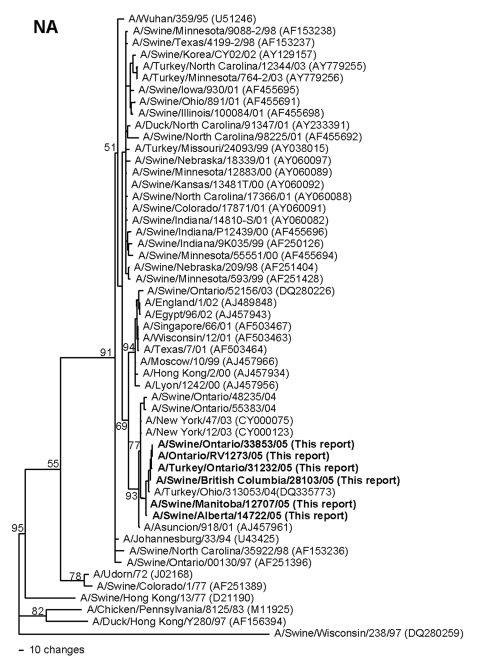
Nucleotide phylogram for the neuraminidase (NA) genes of A/Swine/Manitoba/12707/05, A/Swine/Alberta/14722/05, A/Swine/British Columbia/28103/05, A/Swine/Ontario/33853/05, A/Turkey/Ontario/31232/05, A/Ontario/RV1273/05, and related reference viruses. The evolutionary relationships among these viruses were estimated by the method of maximum parsimony (PAUP software, version 4.0b10) with a bootstrap resampling method (200 replications) and a fast heuristic search algorithm. Numbers at the nodes of the phylograms indicate the bootstrap confidence levels. Horizontal-line distances are proportional to the minimum numbers of nucleotide changes needed to join nodes and gene sequences. Vertical lines are present to space the branches and labels. GenBank accession numbers for the sequences of all reference viruses are provided in parentheses after the virus names.

Pairwise nucleotide identities among the 2005 Canadian swine, turkey, and human isolates range from 94.0%–100% (NA) to 99.9%–100% (M), and amino acid identities range from 98.9%–100% (NA) to 100% (M, NP). The human and swine isolates recovered on a single farm in Ontario are 100% identical in nucleotide sequences across all 8 RNA segments. Phylogenetically, the 2005 Canadian viruses form single clusters on phylograms for each of the 8 viral RNA segments, confirming that this epizootic was caused by a single lineage of viruses. All of the viruses share the same human/classical swine/avian triple reassortant genotype as the H3N2 viruses that emerged in pigs in the United States in 1998 (*2**–**4*). The HA genes (Figure 1) of the Canadian viruses are most closely related to the cluster III group of American viruses that were first isolated from pigs in 1999 (*4*) and subsequently from turkeys (*7**,**8*), though the HA phylogram topography suggests that these Canadian and related US viruses represent a new, separate cluster (IV) of viruses. The M, NP, NS, and polymerase genes of the Canadian viruses are also phylogenetically closely related to reassortant viruses dating back to 1998 in the United States (data not shown). In contrast, the NA genes (Figure 2) of the 2005 Canadian viruses, though still clearly of human lineage, are phylogenetically distinct from most of the US swine and turkey isolates. This lineage is represented by human H3N2 isolates from Asunción, Paraguay, (2001) and New York (2003). However, this lineage of NA genes has also been introduced into animal influenza viruses on 2 previous occasions. The first is represented by A/Turkey/Ohio/313053/04 (*8*), which phylogenetically is the most closely related virus to the Canadian viruses across all 8 RNA segments. Since this virus was isolated in February 2004 (*8*), nearly 1 year before the first isolations of viruses from Canada, one might conjecture that this or a closely related virus from the United States was the source of the Canadian viruses. However, this lineage of NA genes was also already present in human/classical swine reassortant H1N2 viruses (A/Swine/Ontario/48235/04, A/Swine/Ontario/55383/04) isolated from Canadian pigs in 2004 (*12*). Thus, it is neither possible nor prudent from the phylogenetic data alone to define the specific epidemiologic source(s) of the 2005 Canadian H3N2 viruses. However, the appearance of this lineage of NA genes among H3N2 viruses in turkeys in the United States and Canada and H3N2 and H1N2 viruses in pigs in Canada suggests that a complicated web of interspecies transmission, reassortment, and transboundary movement of viruses occurred in a relatively short period of time.

## Conclusions

To our knowledge, this report describes the first isolation of a human/classical swine/avian triple reassortant H3N2 virus from a human. This isolation could not have occurred through cross-contamination in a laboratory since the animal and human virus isolations and sequencing were conducted in different locations. Hemagglutination-inhibition (HI) and virus neutralization (VN) assays of acute- and convalescent-phase (11 and 45 days after the acute-phase sample) sera did not show evidence of seroconversion against the patient's own isolate, A/Ontario/RV1273/05 (HI titer = 8 and VN titer = 16 on all 3 test dates). Thus, although this farm worker had a febrile respiratory illness and no other etiologic agent was identified, we cannot prove that he was actively infected with the triple reassortant virus; he may have simply been harboring the virus in his nasal passages. Nonetheless, this isolation shows that agricultural workers may be exposed to influenza viruses from livestock.

In summary, this report describes the emergence and rapid spread since January 2005 of reassortant H3N2 influenza A viruses among pigs and turkeys across Canada and isolation of a related virus from the nasal passages of a farm worker in Ontario. The 4 swine isolates chosen for our analyses provide a sampling of viruses from British Columbia to Ontario, but clinical reports indicate that the outbreak of ILI in pigs was much more extensive than this limited number of isolates might suggest. For example, H3N2 virus infections were confirmed on 22 swine farms in Ontario between late April and early July 2005. Likewise, additional infections of turkeys with H3 viruses in 2005 were reported in British Columbia (on a farm that was near a swine farm where H3 virus was detected [13]) and in multiple flocks in Manitoba (A. Hamel and G. Nayar, pers. comm.). When this Canadian epizootic is considered together with the extensive spread of genotypically similar H3N2 and H1N2 viruses in pigs and turkeys seen in the United States since 1998, we see that viruses with this human/classical swine/avian triple reassortant genotype can efficiently infect both pigs and turkeys.

The GenBank numbers assigned to the gene sequences of viruses investigated in this study are as follows: A/Ontario/RV1273/05, DQ469955–DQ469962; A/Swine/Alberta/14722/05, DQ469963–DQ469970; A/Swine/British Columbia/28103/05, DQ469971–DQ469978; A/Swine/Manitoba/12707/05, DQ469979–DQ469986; A/Swine/Ontario/33853/05, DQ469987–DQ469994; and A/Turkey/Ontario/31232/05, DQ469995–DQ470002.
